# Activity of Antibiotics and Potential Antibiofilm Agents against Biofilm-Producing *Mycobacterium avium*-*intracellulare* Complex Causing Chronic Pulmonary Infections

**DOI:** 10.3390/antibiotics11050589

**Published:** 2022-04-27

**Authors:** Elena Portell-Buj, Cecibel González-Criollo, Alexandre López-Gavín, Mariana Fernández-Pittol, Maria Antònia Busquets, Joan Estelrich, Montserrat Garrigó, Marc Rubio, Griselda Tudó, Julian Gonzalez-Martin

**Affiliations:** 1 Departament de Fonaments Clínics, Facultat de Medicina i Ciències de la Salut, Universitat de Barcelona, c/Casanova 143, 08036 Barcelona, Spain; elenapb_92@hotmail.com (E.P.-B.); cecigc83@yahoo.es (C.G.-C.); axlz1990@gmail.com (A.L.-G.); mjfernandez@clinic.cat (M.F.-P.); griselda.tudo@ub.edu (G.T.); 2 ISGlobal Barcelona, Institute for Global Health, c/Rosselló 132, 08036 Barcelona, Spain; 3 Servei de Microbiologia, CDB, Hospital Clínic de Barcelona, c/Villarroel 170, 08036 Barcelona, Spain; 4 Unidad de Investigación en Biomedicina, Zurita & Zurita Laboratorios, Quito 170104, Ecuador; 5 Department de Farmàcia, Tecnologia Farmacèutica i Físicoquímica, Facultat de Farmàcia i Ciències de l’Alimentació, Universitat de Barcelona, Av. Joan XXIII, 27-31, 08028 Barcelona, Spain; mabusquetsvinas@ub.edu (M.A.B.); joanestelrich@ub.edu (J.E.); 6 Institut de Nanociència i Nanotecnologia, IN2UB, Facultat de Química, Av. Diagonal 645, 08028 Barcelona, Spain; 7 Servei de Microbiologia, Fundació de Gestió de l’Hospital de la Santa Creu i Sant Pau, c/Sant Quintí 89, 08026 Barcelona, Spain; mgarrigo@santpau.cat; 8 Institut d’Investigació Biomèdica Sant Pau (IIB Sant Pau), c/Sant Quintí 77, 08041 Barcelona, Spain; mrubiobu@santpau.cat; 9 CIBER of Infectiuos Diseases (CIBERINFEC), Instituto de Salud Carlos III, 28029 Madrid, Spain

**Keywords:** nontuberculous mycobacteria, biofilm, antibiotic combinations, potential antibiofilm agents, minimum biofilm inhibitory concentrations, minimum biofilm eradication concentrations

## Abstract

Nontuberculous mycobacteria (NTM) cause lung infections in patients with underlying pulmonary diseases (PD). The *Mycobacterium* *avium*-*intracellulare* complex (MAC) is the most frequently involved NTM. The MAC-PD treatment is based on the administration of several antibiotics for long periods of time. Nonetheless, treatment outcomes remain very poor. Among the factors involved is the ability of MAC isolates to form biofilm. The aim of the study was to assess the in vitro activity of different antibiotics and potential antibiofilm agents (PAAs) against MAC biofilm. Four antibiotics and six PAAs, alone and/or in combination, were tested against planktonic forms of 11 MAC clinical isolates. Biofilm was produced after 4 weeks of incubation and analyzed with the crystal violet assay. The antibiotics and PAAs were tested by measuring the absorbance (minimum biofilm inhibition concentrations, MBICs) and by performing subcultures (minimum biofilm eradication concentrations, MBECs). The clarithromycin/amikacin and clarithromycin/ethambutol combinations were synergistic, decreasing the MBECs values compared to the individual antibiotics. The amikacin/moxifloxacin combination showed indifference. The MBIC values decreased significantly when PAAs were added to the antibiotic combinations. These results suggest that antibiotic combinations should be further studied to establish their antibiofilm activity. Moreover, PAAs could act against the biofilm matrix, facilitating the activity of antibiotics.

## 1. Introduction

Nontuberculous mycobacteria (NTM) include over 200 mycobacterial species other than *Mycobacterium tuberculosis* and *Mycobacterium leprae*. They are broadly found in the environment (e.g., soil and water systems) and are generally non-pathogenic to humans [1]. However, NTM can originate a diverse range of infections, from lung to skin infections, in susceptible individuals, such as immunosuppressed patients and those with pre-existing pulmonary diseases (PD) (e.g., bronchiectasis, cystic fibrosis and chronic obstructive pulmonary disease, COPD). The *Mycobacterium avium*-*intracellulare* complex (MAC) is one of the most clinically significant, slowly growing mycobacteria (SGM) and is frequently involved in NTM-PD [2]. There is currently no standardized treatment against MAC-PD. However, the British Thoracic Society (BTS) guidelines recommend the administration of multiple antibiotics (i.e., rifampicin, ethambutol (EMB), azithromycin and clarithromycin (CLR)) for a minimum of twelve months [3]. Nonetheless, treatment outcomes remain very poor, and reinfection and relapse are remarkably common. Currently, patients suffering from MAC-PD show 8.3–48% of recurrence [4].

MAC, as well as other NTM and *M. tuberculosis*, have the ability to form biofilms [5]. There is little knowledge about the proportion of isolates that can form biofilm and its association to virulence. Nevertheless, non-biofilm forming isolates would have less ability to colonize the human mucosa and tissues [6]. Biofilms are a community of microorganisms embedded in a surface and surrounded by a self-made matrix [7]. They offer protection from the environment, the host immune response and antibiotics, among others. Thus, microorganisms thriving within biofilms display differential traits compared to their planktonic counterparts, such as increased antibiotic tolerance and persistence [8,9]. The antibiotic concentrations needed to eradicate biofilm infections can be several thousand times higher than those required in non-biofilm forming infections [10]. As a result, biofilms appear to be a key virulence factor in chronic infections, making MAC-PD treatment even more difficult [6,11]. However, in microbiology diagnostic laboratories, antibiotic susceptibility testing is routinely performed with planktonic forms. Consequently, the minimum inhibitory concentrations (MIC) determined with common methods cannot predict the antibiotic concentrations needed to eradicate biofilm infections [10].

Given the poor MAC-PD treatment outcomes, it is essential to develop novel treatments that not only target the planktonic forms, but also MAC biofilm. Unfortunately, there are limited clinically available options. However, a few studies have used novel approaches against MAC biofilm by targeting the biofilm matrix [11]. For instance, agents such as Tween 80 and *N*-acetyl-l-cysteine (NALC) have proved to be effective in disrupting the biofilm matrix, and thus improving antibiotic penetration at the site of infection [7]. Different anti-inflammatory drugs have shown to reduce inflammation in animal models of tuberculosis, contributing to a better response to the treatment [12,13]. Therefore, we hypothesized that anti-inflammatory drugs, such as acetyl-salicylic acid (ASA), paracetamol (PCM) and ibuprofen (IBP), could have a lytic activity against the biofilm. We also included diallyl disulphide (DADS), an organosulfur compound derived from garlic that has been associated with biofilm reduction in *Pseudomonas aeruginosa* pulmonary infections. Further research of the MAC-PD biofilm could improve patient management and result in better treatment outcomes. Consequently, the objective of the present work was to study the in vitro activity of different antibiotics and potential antibiofilm agents (PAAs), alone and in combination, against MAC biofilm.

## 2. Results

Table 1 shows the MIC_90_ of the following ten clinical isolates: four clinical isolates of *M. avium* and six clinical isolates of *M. intracellulare*, plus the reference strain *M. avium* ATCC 25291, for four antibiotics (amikacin (AMK), CLR, EMB and moxifloxacin (MXF)), and six PAAs (ASA, DADS, IBP, NALC, PCM and Tween 80). The different antibiotics showed MICs values ranging from 2 µg/mL to 16 µg/mL against the planktonic forms. The PAAs showed MIC values of >64 µg/mL. Table 1 also shows the minimum biofilm eradication concentrations (MBEC)_90_ of the biofilm forming forms (BFF) for all the antibiotics and PAAs tested. All of these showed a MBEC_90_ of 4096 µg/mL.

Table 2 displays the combined inhibitory concentration (CIC)_90_ and the fractional inhibitory concentration index (FICI)_90_ of three two-antibiotic combinations (CLR/AMK, AMK/MXF and CLR/EMB), against all of the 11 isolates (including the reference strain). In the planktonic forms, the fractional inhibitory concentrations (FIC)_90_ of the combinations were indifferent (ranging from 0.75 to 4). However, in BFFs, the combination CLR/AMK was synergistic in *M. avium* and *M. intracellulare* isolates and the combination CLR/EMB was synergistic in *M. intracellulare* and indifferent in *M. avium*. The combination of AMK/MXF was indifferent in both species.

Table 3 shows six three-drug combinations, including two antibiotics (CLR and AMK) and one of the six PAAs against the eleven isolates. The MBECs obtained were 1–2 dilutions lower than those observed with the two-antibiotic combinations. Moreover, the combination CLR/AMK was synergistic, when combined with the PAAs, against *M. intracellulare* isolates. Synergism was observed when combining CLR/AMK with the PAAs, except for NALC and Tween 80 in *M. avium* isolates.

In addition, Table 1, Table 2 and Table 3 show the minimum biofilm inhibitory concentrations (MBIC_90_) in BFFs. An overall correlation was observed between the MBEC_90_ and the MBIC_90_ for the individual antibiotics and for the three two-antibiotics combinations.

The individual results of each clinical isolate are displayed as Supplemental Materials (Tables S1–S5).

## 3. Discussion

In the present work, we studied the in vitro activity of four antibiotics, alone and in combination, against planktonic and BFFs of five *M. avium* and six *M. intracellulare* clinical isolates. The antibiotic combinations were studied with and without PAAs.

The main findings of the study were the synergistic activity of the combinations CLR/AMK against *M. avium* and CLR/AMK and AMK/MXF against *M. intracellulare.* The third combination, CLR/EMB, was indifferent against both species (Table 2). Additionally, we observed synergistic activity when PAAs, such as ASA, IBP, PCM and DADS, were added to the antibiotic combination CLR/AMK (Table 3).

There is no standardized method to study biofilm formation in mycobacteria, hampering the comparison of the results between different studies. However, some methods, such as the adherence in 96-well plates and the crystal violet assay, which were used in the present work, have been described. Moreover, the Calgary Biofilm device and bioreactors have also proved to be effective [14]. In general, biofilm assessment is based on dyeing its structure macroscopically, or using fluorescent dyes and confocal laser scanning microscopy to observe the matrix and the viability of the bacteria within the biofilm.

There is a strong interest in studying the activity of antibiotics against biofilm forming infections. Respiratory infections associated with biofilm formation mainly occur in patients suffering from bronchiectasis and cystic fibrosis and most of the literature is about *P. aeruginosa* and is very scarce regarding mycobacteria. Furthermore, the literature is mainly focused on rapidly growing mycobacteria (RGM). Differences in the antibiofilm activity of antibiotics have been observed in RGM. For instance, Muñoz-Egea et al. [15] observed a higher in vitro activity of ciprofloxacin, when combined with NALC or Tween 80, than CLR or AMK in *Mycobacterium smegmatis* and *Mycobacterium fortuitum*, while in the planktonic forms, the opposite occurred. This fact could be related to the chemical properties of each antibiotic. Moreover, in the presence of biofilm, the concentrations required to eradicate mycobacteria can be up to 100 to 100,000 times higher than the MICs of the planktonic forms. However, the addition of detergents and mucolytics, such as Tween 80 or NALC, enabled the activity of antibiotics and reduced the MBECs in two dilutions [15]. In our study, the MBECs remained high (4096 µg/mL) for all the antibiotics and without significant differences, albeit in this study, ciprofloxacin was not included. Recently, Nguyen et al. [16] analyzed the antibiofilm activity of the imidazole-amines 12j and 12g (4-(4-(pentyloxy) phenyl)-5-(trifluoromethyl)-1H-imidazol-2-amine and 4-(4-hexylphenyl)-5-(trifluoromethyl)-1H-imidazol-2-amine), which in combination with isoniazid and rifampicin, showed synergistic activity against *M. smegmatis* biofilm. In recent years, several antibiofilm agents have been tested against different bacterial species, given that a high percentage of chronic infections are mediated by biofilm formation [17]. In addition, new drug delivery systems and novel substances are being developed to potentiate drug activity, such as the use of silver nanoparticles, with promising antibacterial and antifungal activity [18].

In this paper, three combinations were studied, showing indifferent activity against the planktonic forms with FICs ranging from 1.25 to 2.25 (Table 2). Nonetheless, two of the three combinations showed synergistic activity against BFFs, reducing the MBECs in more than two dilutions and with FICs of ≤ lower than 0.5. The third combination, CLR/EMB, although being indifferent, had better activity than the individual antibiotics. These data show that the two synergistic combinations could have had simultaneous activity against both biofilm and mycobacteria. Moreover, our results, as well as the previously reported data [15,16], highlight the importance of testing antibiotic combinations, which include antibiotics usually administered to treat planktonic forms, as well as others with potential activity against BFF.

Although the synergistic concentrations found are not low enough to be achieved in the bloodstream with the usually administered doses, it highlights the importance of exploring new strategies to treat mycobacteria [19]. For instance, new systems of antibiotic delivery, such as liposomes and nanoparticles. Rose et al. [20] reported that in vitro models of liposomal AMK for inhalation were more successful than free AMK in eradicating *M. avium.* These methods of antibiotic administration reduce side effects, by allowing the sustained delivery of high concentrations directly to the site of infection.

On the other hand, in the present study, six PAAs were also tested. Two of these, NALC and Tween 80, had been previously studied, showing antibiofilm activity against RGM [15]. Additionally, three of these were analgesic/anti-inflammatory drugs, ASA, IBP and PCM. ASA and IBP are known to reduce local inflammation, modulate tissue destruction and the host response against tuberculosis [12,13]. We hypothesized that they could also have antibiofilm activity. Finally, DADS is an agent derived from garlic and was included due to its antibacterial and antibiofilm activity, previously described in *Salmonella tiphimurium* and *P. aeruginosa* [21,22].

Interestingly, when the PAAs were added to the antibiotic combination of CLR/AMK, we observed synergistic activity of the six PAAs against *M. intracellulare* and four of these (ASA, DADS, IBP and PCM) against *M. avium* (Table 3). This was accompanied by a significant reduction in the MBIC (A_580_ values). This fact suggests that these six agents could have activity against the biofilm matrix, and thus enabling the activity of antibiotics. The confirmation of this finding in future studies would allow for the possibility of including these agents in the current treatment compounds addressed to destroy the mycobacterial biofilm.

Lastly, from a methodological perspective, the lack of standardized methods for biofilm analysis is a limitation, given that it can lead to different interpretations and conclusions.

In comparison with the absorbance determination, subculturing may correlate better to the antibiotic activity, as it directly reflects the number of mycobacteria present. Previous studies have used similar methodologies and/or confocal microscopy [7,15].

As a final conclusion, the combination of CLR/AMK displayed synergy against both MAC species. In addition, the combination of AMK/MXF also showed synergistic activity in *M. intracellulare* isolates. A synergistic activity was observed when ASA, IBP, PCM and DADS were added to the CLR/AMK combination in *M. avium* BFF. Regarding *M. intracellulare* BFFs, all the potential antibiofilm agents had synergistic activity when added to the CLR/AMK combination.

## 4. Materials and Methods

### 4.1. Mycobacterium avium-intracellulare Complex Isolates

Four *M. avium* and six *M. intracellulare* clinical isolates obtained from the Microbiology Department of the Hospital Clinic of Barcelona were used in the present study. The ten clinical isolates were obtained from the respiratory samples of ten different patients. All of the isolates had previously shown the ability to form biofilm in vitro. The reference strain *M. avium* ATCC 25,291 was also included.

### 4.2. Antibiotics and Potential Antibiofilm Agents

The four antibiotics were selected among those recommended in the empiric treatment against MAC infections. The three two-drug combinations studied were designed according to the following criteria: CLA-EMB for oral administration; CLA-AMK for the treatment of severe cases and AMK-MOX for macrolide resistant cases. According to the results obtained, the best combination studied included PAAs.

AMK, CLR, EMB and MXF, as well as the PAAs, NALC, Tween 80, ASA, IBP, PCM and DADS, were obtained from Sigma-Aldrich (St. Louis, MO, USA). AMK, EMB, MXF, NALC, Tween 80, ASA and PCM were dissolved in sterile distilled water. CLR was dissolved in acetone and sterile distilled water. IBP was dissolved in dimethyl sulfoxide (DMSO) (final concentration of 0.002%) (Panreac Applichem, Barcelona, Spain) and sterile distilled water. DADS was dissolved in absolute ethanol (Sigma-Aldrich, St. Louis, MO, USA) and sterile distilled water. All the antibiotics and PAAs were sterilized by filtration and stored at −20 °C until use.

**Figure 1:**
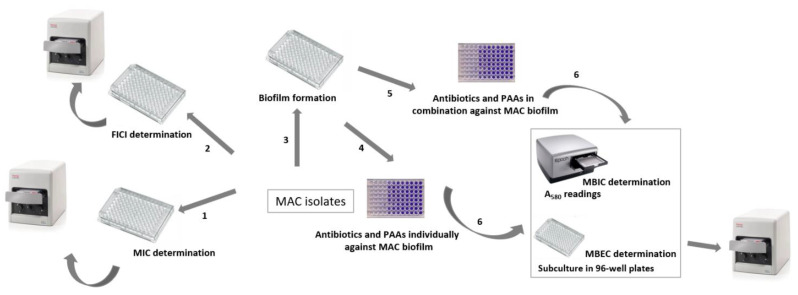
Experimental design for the study of antibiotics and potential antibiofilm agents (PAAs), individually and in combination, against *Mycobacterium avium*-*intracellulare* complex (MAC) producing biofilm. 1. Determination of minimum inhibitory concentration (MIC) with the Vizion System; 2. determination of fractional inhibitory concentration index (FICI) with the Vizion System; 3. biofilm formation; 4–6. determination of minimum biofilm inhibitory concentration (MBIC) and minimum biofilm eradication concentration (MBEC) using the crystal violet assay and subculture, respectively.

The experimental design for the MAC clinical isolates studied is explained in Figure 1.

### 4.3. Minimum Inhibitory Concentration

The MCIs for AMK, CLR, EMB, MXF, NALC, Tween 80, ASA, IBP, PCM and DADS, alone and in combination, were determined in 96-well plates (Smartech Biosciences, Barcelona, Spain). In brief, 100 µL of Middlebrook 7H9 liquid media (Becton Dickinson, Sparks, MD, USA) were added to each well. Then, 100 µL of antibiotic were added to the first well and two-fold serial dilutions, ranging from 64 µg/mL to 0.5 µg/mL, were made. The same procedure and concentrations were used for the PAAs, except for Tween 80, which was tested in dilutions ranging from 12.5% to 0.09%. Finally, 100 µL of inoculum, at a concentration of 1.5 × 10^5^ CFU/mL, were added (1/1000 dilution of a 0.5 McFarland, using a nephelometer) (PhoenixSpec, Becton Dickinson). The positive control wells contained 100 µL of Middlebrook 7H9 and 100 µL of inoculum. The negative control wells were also included, by adding 200 µL of Middlebrook 7H9. The microplates were incubated at 37 °C for 7 days. After incubation, the plates were read using the Vizion System (Sensititre Vizion Digital MIC Viewing System, Thermo Fisher Scientific, Waltham, MA, USA). The MIC value was interpreted as the lowest antibiotic and/or PAA concentration inhibiting mycobacterial growth.

### 4.4. Fractional Inhibitory Concentration Index

MICs, in combination with two antibiotics, were determined by crossing the individual MIC and the two concentrations below the MIC for each antibiotic with the corresponding one of the other antibiotics. This antibiotic interaction was analyzed using the FICI method, as proposed by Den Hollander et al. [23]. The FICI is the addition of the FIC of each antibiotic present in the combination. The FIC was calculated as a quotient between the CIC and the MIC of each drug using the following equation: FICI = FIC_A_ + FIC_B_ = (CIC_A/_MIC_A_) + (CIC_B_/MIC_B_), where the CIC value is the lowest drug concentration that inhibits bacterial growth when the antibiotic acts in combination, and the MIC value is the lowest drug concentration that inhibits bacterial growth when the antibiotic acts individually.

The results of the FICI analysis were interpreted according to the following criteria: a decrease of two dilutions under the individual MIC was interpreted as synergistic with a FICI of <0.5; indifference was determined from 0.5 to 4; and a FICI of >4 was considered as antagonistic activity.

The CIC_90_ and the FICI_90_ values, shown in Table 2 and Table 3, were defined as the values of CIC and FICI that included 90% of the isolates tested.

### 4.5. Biofilm Formation

The in vitro biofilm was formed as previously described [24]. Briefly, the isolates were grown in Middlebrook 7H9 broth. Then, the mycobacterial cultures were homogenised by agitation and adjusted to a concentration of 1 × 10^7^ CFU/mL, using a nephelometer. Afterwards, 200 µL of inoculum (1 × 10^7^ CFU/mL) were seeded in each well of non-treated polystyrene plates (Thermo Fisher Scientific, Waltham, MA, USA). The plates were incubated for 4 weeks at 42 °C in the case of *M. avium* and at 37 °C for *M. intracellulare* (see Supplemental Data; Figure S1A) Negative controls containing 200 µL of Middlebrook 7H9 were also included. In order to minimize evaporation, sterile distilled water was added to the surrounding well and the plates were covered with a lid. Each isolate was studied in duplicate in different plates.

### 4.6. Minimum Biofilm Eradication Concentrations and Minimum Biofilm Inhibition Concentrations

After incubation for the biofilm formation, the plates were treated with different antibiotics and PAAs, by adding in each well 100 µL of the desired drug at concentrations ranging from 4096 µg/mL to 32 µg/mL, except for Tween 80, which was tested in dilutions ranging from 12.5% to 0.09%. The plates were incubated again for another week at 37 °C. Then, the supernatant of the plates was discarded, and each well was rinsed once with 200 µL of 1× phosphate-buffered saline (PBS) (see Supplemental Data; Figure S1B). The plates were dried at 60 °C for 1 h and the wells were dyed with 200 µL of 1% crystal violet. The plates were incubated at room temperature for 10 min and blotted on paper towels. Each well was rinsed once with 200 µL of 1× PBS and dried at 60 °C for 1 h. Then, 200 µL of 33% acetic acid were added in order to solubilize the biofilm (see Supplemental Data; Figure S1C). Finally, the A_580_ was determined using a microplate spectrophotometer (BioTek Instruments Inc., Winooski, VT, USA). The wells containing only Middlebrook 7H9 medium, as well as only antibiotics or PAAs, were used as blanks and their mean A_580_ values were subtracted from the wells containing biofilm. The A_580_ values from the wells without antibiotics and/or PAAs were considered 100%. The wells containing antibiotics, individually or in combination, and/or PAAs were interpreted as a percentage of these values. In the present study, the MBIC was defined as the lowest concentration of antibiotic and/or PAA, decreasing by ≥40% the A_580_ value [25,26]. The MBEC_90,_ values were defined as the lowest concentration that decreased by ≥40% the A_580_ value in 90% of the isolates, respectively.

Before the A_580_ readings, subcultures of the plates were made in a second plate, by seeding 20 µL of each well in a new well containing 180 µL of Middlebrook 7H9. These plates were then incubated for 1 week at 37 °C. After incubation, the plates were checked for visual growth using the Vizion System.

The MBEC was defined as the lowest concentration of antibiotic, individually or in combination, and/or PAA inhibiting biofilm formation. The MBEC_90,_ values were defined as the lowest concentration that eradicated the biofilm formation in 90% of the isolates. The calculation of FIC and FICI for biofilm formation analysis was performed as previously described for the planktonic forms.

## 5. Conclusions

The main conclusion of the present study is that antibiotic combinations are significantly better than individual antibiotics against BFF. Furthermore, the addition of PAAs to antibiotic combinations could increase their activity. The results lead the path for further studies with other combinations, including common and new antibiotics, as well as potential antibiofilm agents, as tested in this study. These data could also open new therapeutic options based on the clinical use of these compounds, particularly in inhaled formulations, by feasibly administering higher doses with lower side effects.

## Figures and Tables

**Table 1:** MICs, MBECs and MBICs of the antibiotics and potential antibiofilm agents, tested against planktonic and BFFs of 4 *M. avium* and 6 *M. intracellulare* clinical isolates and the reference strain *M. avium* ATCC25291.

**Isolates**	**MICs of Planktonic Forms (µg/mL)**									
	**AMK**	**CLR**	**EMB**	**MXF**	**ASA**	**DADS**	**IBP**	**NALC**	**PCM**	**Tween 80**
*M. avium*										
MIC_90_	16	4	8	2	>64	>64	>64	>64	>64	>64
*M. intracellulare*										
MIC_90_	16	2	8	2	>64	>64	>64	>64	>64	>64
*M. avium* ATCC										
MIC	8	2	4	1	>64	>64	>64	>64	>64	>64
**Isolates**	**MBECs and MBICs of Biofilm Forming Forms (µg/mL)**									
	**AMK**	**CLR**	**EMB**	**MXF**	**ASA**	**DADS**	**IBP**	**NALC**	**PCM**	**Tween 80**
*M. avium*										
MBEC_90_	4096	4096	4096	4096	4096	4096	4096	4096	4096	4096
MBIC_90_	2048	2048	2048	4096	4096	4096	4096	4096	4096	4096
*M. intracellulare*										
MBEC_90_	4096	4096	4096	4096	4096	4096	4096	4096	4096	4096
MBIC_90_	4096	4096	2048	1024	4096	4096	4096	4096	4096	4096
*M. avium* ATCC										
MBEC	1024	512	2048	256	4096	4096	4096	4096	4096	4096
MBIC	1024	256	256	512	4096	4096	4096	4096	4096	4096

MIC: minimum inhibitory concentration; MBEC: minimum biofilm eradication concentration; MBIC: minimum biofilm inhibitory concentration; CLR: clarithromycin; AMK: amikacin; MXF: moxifloxacin; EMB: ethambutol; ASA: acetyl-salicylic acid; IBP: ibuprofen; PCM: paracetamol; NALC: *N*-acetyl-l-cysteine; DADS: diallyl disulphide.

**Table 2:** CICs, FICIs, MBECs and MBICs of the antibiotic combinations against planktonic and BFFs of 4 *M. avium* and 6 *M. intracellulare* clinical isolates and the reference strain *M. avium* ATCC25291.

**Isolates**	**CICs and FICIs of Planktonic Forms (µg/mL)**					
	**CIC_90_** **CLR/AMK**	**FICI_90_** **CLR/AMK**	**CIC_90_** **AMK/MXF**	**FICI_90_** **AMK/MXF**	**CIC_90_** **CLR/EMB**	**FICI_90_** **CLR/EMB**
*M. avium*	4	2.25	4	2.5	4	2.5
*M. intracellulare*	4	2.5	4	2.25	4	3
*M. avium* ATCC	4	2.5	2	2.25	4	3
**Isolates**	**MBECs and MBICs of Biofilm Forming Forms (µg/mL)**					
	**CLR/AMK**	**FICI_90_** **CLR/AMK**	**AMK/MXF**	**FICI_90_** **AMK/MXF**	**CLR/EMB**	**FICI_90_** **CLR/EMB**
*M. avium*						
MBEC_90_	256	0.25 *	256	1.12	512	0.5
MBIC_90_	1024	ND	2048	ND	2048	ND
*M. intracellulare*						
MBEC_90_	512	0.25 *	512	0.25 *	512	0.75
MBIC_90_	512	ND	2048	ND	512	ND
*M. avium* ATCC						
MBEC	256	0.75	256	1.12	256	0.625
MBIC	1024	ND	1024	ND	2048	ND

AMK: amikacin; CLR: clarithromycin; EMB: ethambutol; MXF: moxifloxacin; CIC: minimum combined inhibitory concentration; FICI: fractional inhibitory concentration index; MBEC: minimum biofilm eradication concentration; MBIC: minimum biofilm inhibitory concentration; ND: not done. * Synergistic activity.

**Table 3:** MBECs and MBICs of the combinations including antibiotics and potential antibiofilm agents, tested against BFFs of 4 *M. avium* and 6 *M. intracellulare* clinical isolates and the reference strain *M. avium* ATCC25291.

Isolates	MBIC (µg/mL)											
	C/K/A	FICI_90_ C/K/A	C/K/D	FICI_90_ C/K/D	C/K/I	FICI_90_ C/K/I	C/K/N	FICI_90_ C/K/N	C/K/P	FICI_90_ C/K/P	C/K/T80	FICI_90_ C/K/T80
*M. avium*												
MBEC_90_	128	0.15 *	128	0.3 *	128	0.15 *	256	0.3 *	128	0.15 *	256	0.3 *
MBIC_90_	128	ND	128	ND	128	ND	256	ND	128	ND	256	ND
*M. intracellulare*												
MBEC_90_	128	0.12 *	128	0.0.9 *	256	0.18 *	128	0.18 *	256	0.37	256	0.18 *
MBIC_90_	128	ND	128	ND	256	ND	128	ND	256	ND	256	ND
*M. avium* ATCC												
MBEC_90_	128	0.40	128	0.40	128	0.40	256	0.81	128	0.40	256	0.81
MBIC_90_	128	ND	128	ND	128	ND	256	ND	128	ND	512	ND

MBEC: minimum biofilm eradication concentration; MBIC: minimum biofilm inhibitory concentration; FICI: fractional inhibitory concentration index; C: clarithromycin; K: amikacin; A: acetyl-salicylic acid; D: diallyl disulphide; I: ibuprofen; N: *N*-acetyl-l-cysteine; P: paracetamol; T80: Tween 80; ND: not done. * Synergistic activity.

## Data Availability

Data is contained within the article.

## Supplementary Materials

The following supporting information can be downloaded at: https://www.mdpi.com/article/10.3390/antibiotics11050589/s1, Figure S1: Biofilm formation and the Crystal Violet method used to determine the quantification of the biofilm by measuring the optical density (OD) of the dyed biofilm. (A) Biofilm formation at 37 °C for 4 weeks in Middlebrook 7H9 liquid media; (B) Fixed biofilm at the bottom of the wells after being washed with PBS and dried at 60 °C; (C) Crystal violet dyed, washed and dried biofilm to be read the OD with acetic acid solution; Table S1: Minimum inhibitory concentration of the antibiotics and potential antibiofilm tested, individually and in combination, against 4 *M. avium* and 6 *M. intracellulare* clinical isolates, and the ATCC25291 reference strain; Table S2: Minimum biofilm eradication concentration of antibiotics and potential antibiofilm agents tested, individually and in combination, against 4 *M. avium* and 6 *M. intracellulare* clinical isolates (I), and the ATCC25291 reference strain; Table S3: Minimum biofilm eradication concentration of antibiotics and potential antibiofilm agents tested, individually and in combination, against 4 *M. avium* and 6 *M. intracellulare* clinical isolates (II), and the ATCC25291 reference strain; Table S4: Minimum biofilm inhibitory concentration of the antibiotics and potential antibiofilm agents tested, individually and in combination, against 4 *M. avium* and 6 *M. intracellulare* clinical isolates (I), and the ATCC25291 reference strain; Table S5: Minimum biofilm inhibitory concentration of the antibiotics and potential antibiofilm agents tested, individually and in combination, against 4 *M. avium* and 6 *M. intracellulare* clinical isolates (II), and the ATCC25291 reference strain.
