# Supplementary material for: Activity of Antibiotics and Potential Antibiofilm Agents against Biofilm-Producing Mycobacterium avium-intracellulare Complex Causing Chronic Pulmonary Infections
Source: Antibiotics (Basel). 2022 Apr 27;11(5):589. doi: 10.3390/antibiotics11050589 (PMC9137467; doi:10.3390/antibiotics11050589)
Supplement: Supplementary file 1 [file antibiotics-11-00589-s001.zip › antibiotics-1694715-supplementary.pdf]

Supplementary Materials

Table S1: Minimum inhibitory concentration of the antibiotics and potential antibiofilm tested, individually and in combination, against 4 *M. avium* and 6 *M. intracellulare* clinical isolates, and the ATCC25291 reference strain.

Isolate	MIC (µg/mL)															
	K	C	E	M	C/K	∑FIC C/K	K/M	∑FIC K/M	C/E	∑FIC C/E	A	D	I	N	P	T 80
M. avium 1	16	4	4	1	4	1.25	2	2.12	4	2	>64	>64	>64	>64	>64	>64
M. avium 2	8	4	8	2	4	1.5	4	2.5	4	1.5	>64	>64	>64	>64	>64	>64
M. avium 3	16	4	4	1	4	1.25	2	2.12	4	2	>64	>64	>64	>64	>64	>64
M. avium 4	16	2	8	2	4	2.25	4	2.25	4	2.5	>64	>64	>64	>64	>64	>64
MIC_{90}	16	4	8	2	4	2.25	4	2.5	4	2.5	>64	>64	>64	>64	>64	>64
M. avium ATCC25291	8	2	4	1	4	2.5	2	2.25	4	3	>64	>64	>64	>64	>64	>64
M. intracellulare 1	8	2	4	2	4	2.5	2	1.25	4	3	>64	>64	>64	>64	>64	>64
M. intracellulare 2	16	2	8	2	4	2.25	4	2.25	4	2.5	>64	>64	>64	>64	>64	>64
M. intracellulare 3	8	1	4	1	2	2.25	2	2.25	2	2.5	>64	>64	>64	>64	>64	>64
M. intracellulare 4	8	2	8	1	2	1.25	2	2.25	2	1.25	>64	>64	>64	>64	>64	>64
M. intracellulare 5	8	1	8	1	2	2.25	1	1.125	2	2.25	>64	>64	>64	>64	>64	>64
M. intracellulare 6	8	1	8	1	2	2.25	2	1.25	2	2.25	>64	>64	>64	>64	>64	>64
MIC_{90}	16	2	8	2	4	2.5	4	2.25	4	3	>64	>64	>64	>64	>64	>64

MIC: minimum inhibitory concentration; K: amikacin; C: clarithromycin; E: ethambutol; M: moxifloxacin; A: acetyl-salicylic acid; DADS:diallyl disulphide; I: ibuprofen; N: N-Acetyl-L-cysteine; P: paracetamol; T80: Tween 80.

Table S2: Minimum biofilm eradication concentration of antibiotics and potential antibiofilm agents tested, individually and in combination, against 4 *M. avium* and 6 *M. intracellulare* clinical isolates (I), and the ATCC25291 reference strain.

Isolate	MBECs (µg/mL)															
	K	C	E	M	C/K	∑FIC C/K	K/M	∑FIC K/M	C/E	∑FIC C/E	A	D	I	N	P	T80
M. avium 1	4096	4096	4096	4096	256	0.125	128	0.0625	512	0.25	4096	4096	4096	4096	4096	4096
M. avium 2	2048	2048	2048	256	256	0.25	128	0.5625	512	0.5	4096	4096	4096	4096	4096	4096
M. avium 3	2048	2048	2048	256	256	0.25	256	1.125	256	0.25	4096	4096	4096	4096	4096	4096
M. avium 4	2048	2048	2048	4096	256	0.25	256	0.187	512	0.50	4096	4096	4096	4096	4096	4096
$MBEC_{90}$	4096	4096	4096	4096	256	0.25	256	1.12	512	0.5	4096	4096	4096	4096	4096	4096
M. avium ATCC25291	1024	512	2048	256	256	0.75	256	1.125	256	0.625	4096	4096	4096	4096	4096	4096
M. intracellulare 1	4096	1024	4096	4096	512	0.625	256	0.125	128	0.15625	4096	4096	4096	4096	4096	4096
M. intracellulare 2	4096	2048	1024	1024	256	0.1875	256	0.3125	512	0.75	4096	4096	4096	4096	4096	4096
M. intracellulare 3	4096	4096	4096	4096	512	0.25	512	0.25	128	0.0625	4096	4096	4096	4096	4096	4096
M. intracellulare 4	4096	4096	4096	4096	256	0.125	512	0.25	256	0.125	4096	4096	4096	4096	4096	4096
M. intracellulare 5	4096	4096	2048	1024	128	0.0625	256	0.3125	128	0.09375	4096	4096	4096	4096	4096	4096
M. intracellulare 6	4096	4096	2048	4096	128	0.0625	512	0.25	128	0.09375	4096	4096	4096	4096	4096	4096
$MBEC_{90}$	4096	4096	4096	4096	512	0.25	4096	2	512	0.75	4096	4096	4096	4096	4096	4096

MBEC: minimum biofilm eradication concentration; MBIC: minimum biofilm inhibitory concentration; K: amikacin; C: clarithromycin; E: ethambutol; M: moxifloxacin; A: acetyl-salicylic acid; D: diallyl disulphide; I:ibuprofen; N: N-Acetyl-L-cysteine; P: paracetamol; T80: Tween 80.

Table S3: Minimum biofilm eradication concentration of antibiotics and potential antibiofilm agents tested, individually and in combination, against 4 *M. avium* and 6 *M. intracellulare* clinical isolates (II), and the ATCC25291 reference strain.

Isolate	MBEC (µg/mL)											
	C/K/A	∑FIC C/K/A	C/K/I	∑FIC C/K/I	C/K/P	∑FIC C/K/P	C/K/N	∑FIC C/K/N	C/K/D	∑FIC C/K/D	C/K/T80	∑FIC C/K/T80
M. avium 1	128	0.09375	128	0.09375	128	0.09375	128	0.09375	128	0.09375	256	0.1875
M. avium 2	128	0.15625	128	0.15625	128	0.15625	256	0.3	128	0.15625	256	0.3
M. avium 3	128	0.15625	128	0.15625	128	0.15625	256	0.3	128	0.3	128	0.15625
M. avium 4	128	0.15625	128	0.15625	128	0.15625	256	0.3	128	0.3	128	0.3
$MBEC_{90}$	128	0.15625	128	0.15625	128	0.15625	256	0.3	128	0.3	256	0.3
M. avium ATCC25291	128	0.40625	128	0.40625	128	0.40625	256	0.8125	128	0.40625	256	0.8125
M. intracellulare 1	128	0.1875	32	0.046875	256	0.375	128	0.1875	32	0.046875	256	0.375
M. intracellulare 2	128	0.125	128	0.125	128	0.125	128	0.125	64	0.0625	256	0.25
M. intracellulare 3	128	0.09375	64	0.046875	128	0.09375	128	0.09375	128	0.09375	256	0.1875
M. intracellulare 4	128	0.09375	256	0.1875	128	0.09375	128	0.09375	128	0.09375	128	0.09375
M. intracellulare 5	128	0.09375	256	0.1875	128	0.09375	128	0.09375	128	0.09375	128	0.09375
M. intracellulare 6	128	0.09375	256	0.1875	128	0.09375	128	0.09375	128	0.09375	128	0.09375
$MBEC_{90}$	128	0.125	256	0.1875	256	0.375	128	0.1875	128	0.09375	256	0.375

MBEC: minimum biofilm eradication concentration; C: clarithromycin; K: amikacin; E: ethambutol; A: acetyl-salicylic acid; I: ibuprofen; P: paracetamol; N: N-Acetyl-L-cysteine; D: diallyl disulphide; T80: Tween80.

Table S4: Minimum biofilm inhibitory concentration of the antibiotics and potential antibiofilm agents tested, individually and in combination, against 4 *M. avium* and 6 *M. intracellulare* clinical isolates (I), and the ATCC25291 reference strain.

Isolate	MBICs (µg/mL)															
	K	C	E	M	C/K	∑FIC C/K	K/M	∑FIC K/M	C/E	∑FIC C/E	A	D	I	N	P	T80
M. avium 1	2048	4096	2048	4096	256	ND	2048	ND	512	ND	4096	4096	4096	4096	4096	4096
M. avium 2	1024	128	1024	256	512	ND	512	ND	512	ND	4096	4096	4096	4096	4096	4096
M. avium 3	256	256	128	256	512	ND	256	ND	2048	ND	4096	4096	4096	4096	4096	4096
M. avium 4	256	1024	2048	2014	1024	ND	512	ND	1024	ND	4096	4096	4096	4096	4096	4096
$MBIC_{90}$	2048	4096	2048	4096	1024	ND	2048	ND	2048	ND	4096	4096	4096	4096	4096	4096
M. avium ATCC25291	1024	256	2048	512	1024	ND	1024	ND	2048	ND	4096	4096	4096	4096	4096	4096
M. intracellulare 1	4096	1024	2048	512	512	ND	2048	ND	512	ND	4096	4096	4096	4096	4096	4096
M. intracellulare 2	256	256	512	512	512	ND	256	ND	512	ND	4096	4096	4096	4096	4096	4096
M. intracellulare 3	2048	256	256	1024	512	ND	256	ND	128	ND	4096	4096	4096	4096	4096	4096
M. intracellulare 4	2048	256	512	256	256	ND	512	ND	256	ND	4096	4096	4096	4096	4096	4096
M. intracellulare 5	512	4096	512	512	512	ND	1024	ND	512	ND	4096	4096	4096	4096	4096	4096
M. intracellulare 6	2048	512	512	512	512	ND	512	ND	512	ND	4096	4096	4096	4096	4096	4096
$MBIC_{90}$	4096	4096	2048	1024	512	ND	2048	ND	512	ND	4096	4096	4096	4096	4096	4096

MBIC: minimum biofilm inhibitory concentration; K: amikacin; C: clarithromycin; E: ethambutol; A: acetyl-salicylic acid; D: diallyldisulphide; I: ibuprofen; N: N-Acetyl-L-cysteine; T P: paracetamol; T80: Tween80.

Table S5: Minimum biofilm inhibitory concentration of the antibiotics and potential antibiofilm agents tested, individually and in combination, against 4 *M. avium* and 6 *M. intracellulare* clinical isolates (II), and the ATCC25291 reference strain.

Isolate	MBIC (µg/mL)											
	C/K/A	∑FIC C/K/A	C/K/I	∑FIC C/K/I	C/K/P	∑FIC C/K/P	C/K/N	∑FIC C/K/N	C/K/D	∑FIC C/K/D	C/K/T80	∑FIC C/K/T80
M. avium 1	28	ND	128	ND	128	ND	128	ND	128	ND	256	ND
M. avium 2	28	ND	128	ND	128	ND	256	ND	128	ND	256	ND
M. avium 3	128	ND	128	ND	128	ND	256	ND	128	ND	128	ND
M. avium 4	128	ND	128	ND	128	ND	256	ND	128	ND	128	ND
$MBIC_{90}$	128	ND	128	ND	128	ND	256	ND	128	ND	256	ND
M. avium ATCC25291	128	ND	128	ND	128	ND	256	ND	128	ND	512	ND
M. intracellulare 1	128	ND	128	ND	256	ND	64	ND	64	ND	256	ND
M. intracellulare 2	128	ND	128	ND	128	ND	128	ND	64	ND	256	ND
M. intracellulare 3	128	ND	64	ND	128	ND	128	ND	128	ND	256	ND
M. intracellulare 4	128	ND	256	ND	128	ND	64	ND	128	ND	128	ND
M. intracellulare 5	128	ND	256	ND	128	ND	128	ND	128	ND	128	ND
M. intracellulare 6	128	ND	256	ND	128	ND	128	ND	128	ND	128	ND
$MBIC_{90}$	128	ND	256	ND	256	ND	128	ND	128	ND	256	ND

MBIC: minimum biofilm inhibitory concentration; C: clarithromycin; K: amikacin; E: ethambutol; A: acetyl-salicylic acid; I: ibuprofen; P: paracetamol; N: N-Acetyl-L-cysteine; D: diallyl disulphide; T80: Tween80.

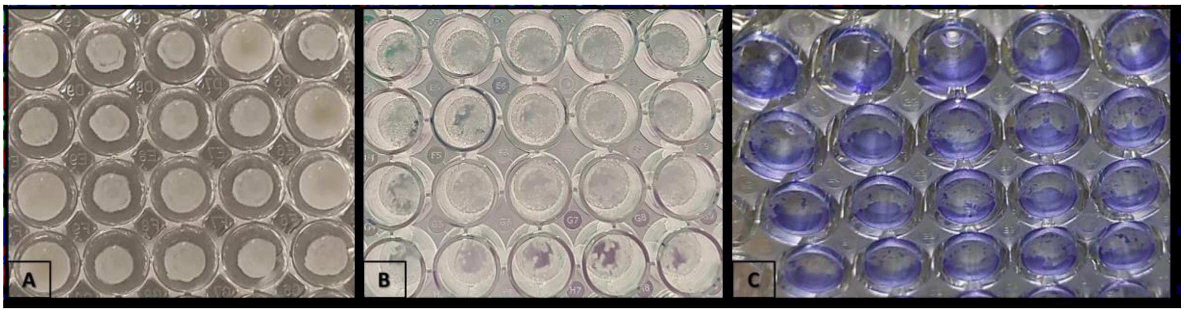

Figure S1: Biofilm formation and the Crystal Violet method used to determine the quantification of the biofilm by measuring the optical density (OD) of the dyed biofilm. A) Biofilm formation at 37 ºC for 4 weeks in Middlebrook 7H9 liquid media; B) Fixed biofilm at the bottom of the wells after being washed with PBS and dried at 60 ºC; C) Crystal violet dyed, washed and dried biofilm to be read the OD with acetic acid solution.
